# Supplementary material for: OpenMM 7: Rapid development of high performance algorithms for molecular dynamics
Source: PLoS Comput Biol. 2017 Jul 26;13(7):e1005659. doi: 10.1371/journal.pcbi.1005659 (PMC5549999; doi:10.1371/journal.pcbi.1005659)
Supplement: S1 Source Code — (ZIP) [file pcbi.1005659.s002.zip › OpenMM-7.0.1-Source/docs/api-c++/generated/OpenMM.DrudeSCFIntegrator.html]

DrudeSCFIntegrator — OpenMM @OPENMM\_MAJOR\_VERSION@.@OPENMM\_MINOR\_VERSION@ documentation


# DrudeSCFIntegrator¶

*class* `OpenMM::``DrudeSCFIntegrator`¶
:   This is a leap-frog Verlet `Integrator` that simulates systems with Drude particles. It uses the self-consistent field (SCF) method: at every time step, the positions of Drude particles are adjusted to minimize the potential energy.

    This `Integrator` requires the `System` to include a `DrudeForce`, which it uses to identify the Drude particles.

    Methods

    |  |  |
    | --- | --- |
    | `DrudeSCFIntegrator` | Create a `DrudeSCFIntegrator`. |
    | `getMinimizationErrorTolerance` | Get the error tolerance to use when minimizing the potential energy. |
    | `setMinimizationErrorTolerance` | Set the error tolerance to use when minimizing the potential energy. |
    | `step` | Advance a simulation through time by taking a series of time steps. |

    `DrudeSCFIntegrator`(double *stepSize*)¶
    :   Create a `DrudeSCFIntegrator`.

        |  |  |
        | --- | --- |
        | Parameters: |  |

        - **stepSize** – the step size with which to integrator the system (in picoseconds)

    double `getMinimizationErrorTolerance`() *const*¶
    :   Get the error tolerance to use when minimizing the potential energy. This roughly corresponds to the maximum allowed force magnitude on the Drude particles after minimization.

        |  |  |
        | --- | --- |
        | Returns: | the error tolerance to use, measured in kJ/mol/nm |

    void `setMinimizationErrorTolerance`(double *tol*)¶
    :   Set the error tolerance to use when minimizing the potential energy. This roughly corresponds to the maximum allowed force magnitude on the Drude particles after minimization.

        |  |  |
        | --- | --- |
        | Parameters: |  |

        - **tol** – the error tolerance to use, measured in kJ/mol/nm

    void `step`(int *steps*)¶
    :   Advance a simulation through time by taking a series of time steps.

        |  |  |
        | --- | --- |
        | Parameters: |  |

        - **steps** – the number of time steps to take

High performance molecular simulation on GPUs


### Navigation

- Core Objects
- Forces
- Integrators
- Extras

©2015, Stanford University and the Authors.
|
Powered by Sphinx 1.3.1
& Alabaster 0.7.7
|
Page source
